# Predictors of Hepatitis B Cure Using Gene Therapy to Deliver DNA Cleavage Enzymes: A Mathematical Modeling Approach

**DOI:** 10.1371/journal.pcbi.1003131

**Published:** 2013-07-04

**Authors:** Joshua T. Schiffer, Dave A. Swan, Daniel Stone, Keith R. Jerome

**Affiliations:** 1 Vaccine and Infectious Diseases Division, Fred Hutchinson Cancer Research Center, Seattle, Washington, United States of America; 2 Department of Medicine, University of Washington, Seattle, Washington, United States of America; 3 Department of Laboratory Medicine, University of Washington, Seattle, Washington, United States of America; John Hopkins University, United States of America

## Abstract

Most chronic viral infections are managed with small molecule therapies that inhibit replication but are not curative because non-replicating viral forms can persist despite decades of suppressive treatment. There are therefore numerous strategies in development to eradicate all non-replicating viruses from the body. We are currently engineering DNA cleavage enzymes that specifically target hepatitis B virus covalently closed circular DNA (HBV cccDNA), the episomal form of the virus that persists despite potent antiviral therapies. DNA cleavage enzymes, including homing endonucleases or meganucleases, zinc-finger nucleases (ZFNs), TAL effector nucleases (TALENs), and CRISPR-associated system 9 (Cas9) proteins, can disrupt specific regions of viral DNA. Because DNA repair is error prone, the virus can be neutralized after repeated cleavage events when a target sequence becomes mutated. DNA cleavage enzymes will be delivered as genes within viral vectors that enter hepatocytes. Here we develop mathematical models that describe the delivery and intracellular activity of DNA cleavage enzymes. Model simulations predict that high vector to target cell ratio, limited removal of delivery vectors by humoral immunity, and avid binding between enzyme and its DNA target will promote the highest level of cccDNA disruption. Development of de novo resistance to cleavage enzymes may occur if DNA cleavage and error prone repair does not render the viral episome replication incompetent: our model predicts that concurrent delivery of multiple enzymes which target different vital cccDNA regions, or sequential delivery of different enzymes, are both potentially useful strategies for avoiding multi-enzyme resistance. The underlying dynamics of cccDNA persistence are unlikely to impact the probability of cure provided that antiviral therapy is given concurrently during eradication trials. We conclude by describing experiments that can be used to validate the model, which will in turn provide vital information for dose selection for potential curative trials in animals and ultimately humans.

## Author Summary

Innovative new approaches are being developed to eradicate viral infections that until recently were considered incurable. We are interested in engineering DNA cleavage enzymes that can cut and incapacitate persistent viruses. One hurdle is that these enzymes must be delivered to infected cells as genes within viral vectors that are not harmful to humans. In this paper, we developed a series of equations that describe the delivery of these enzymes to their intended targets, as well the activity of DNA cutting within the cell. While our mathematical model is catered towards hepatitis B virus infection, it is widely applicable to other infections such as HIV, as well as oncologic and metabolic diseases characterized by aberrant gene expression. Certain enzymes may bind DNA more avidly than others, while different enzymes may also bind cooperatively if targeted to different regions of viral DNA. We predict that such enzymes, if delivered efficiently to a high proportion of infected cells, will be critical to increase the probability of cure. We also demonstrate that our equations will serve as a useful tool for identifying the most important features of a curative regimen, and ultimately for guiding clinical trial dosing schedules to ensure hepatitis B eradication with the smallest number of possible doses.

## Introduction

To date, cure of most chronic viral infections has remained an impossible goal. Replicating forms of hepatitis B virus (HBV), Herpes Simplex Virus (HSV) and Human Immunodeficiency Virus (HIV) can be targeted with potent small molecule therapies, thereby decreasing the burden of disease associated with these pathogens [1]–[4]. However, latent, non-replicating viral genomes persist within reservoirs for each of these infections, and high levels of viral replication typically resume soon after cessation of antiviral therapy, even after years of treatment [5]–[8]. Lifelong therapy is therefore often required, resulting in enormous costs to the healthcare system [9]. In addition, therapy can be complicated by lack of compliance, drug toxicity and resistance.

Curative approaches to these infections will need to target persistent, non-replicating viral genomes. DNA cleavage enzymes, including homing endonucleases (HE) or meganucleases, zinc-finger nucleases (ZFN), transcription activator-like (TALEN) effector nucleases and CRISPR-associated system 9 (cas9) proteins represent a promising new therapeutic approach for targeting these viral forms [10]. These enzymes can be designed to target specific segments of either episomal DNA for HBV and HSV, or integrated viral DNA for HIV, which are vital for replication [11], [12]. When viral DNA is cleaved, it is quickly repaired, allowing for repeated binding of the cleavage enzyme. DNA repair occurs by non-homologous end joining (NHEJ), an error prone process. The enzyme binds to the target site if no mutation occurs during repair. Eventually, target DNA incurs a deletion or insertion that prevents subsequent enzyme binding as well as translation of essential viral proteins. The remaining viral DNA is thereby rendered replication incompetent.

Zinc-finger nucleases are currently being used successfully *ex vivo* as a tool to modify the HIV entry receptors CCR5 and CXCR4 on CD4+ T-cells; altered cells which are resistant to HIV entry or replication have been transplanted back to infected animals as a form of adaptive immunotherapy with resultant decreases in viral load [13], [14], and this method is being tested in human clinical trials [15]. Similar modification of CCR5 in hematopoietic stem cells may allow reconstitution of the full immune system with exclusively HIV-resistant cells [16]. In contrast, our approach here is to use DNA cleavage enzymes that directly target latent viral genomes, rather than host cell viral entry receptors, to be delivered to infected cells as transgenes within viral vectors [10], [17]. However, numerous fundamental questions remain regarding such a gene therapy approach: which vectors are most appropriate for gene delivery? How many doses will be necessary for viral eradication? Can vector delivery be limited to decrease the probability of toxicity? If multiple doses are needed, how will immunity to the delivery vector impact the likelihood of cure? Does reconstitution of latently infected cells occur rapidly enough to necessitate a narrow interval between successive gene therapy doses? Is there benefit to delivering multiple transgenes per vector and should enzymes be engineered to target several regions of viral DNA [10]?

Mathematical models are crucial tools for identifying dynamics of active infection, and for designing antiviral regimens that maximize potency and avoid drug resistance [18], [19]. However, despite twenty years of experience with antiretroviral therapy, a unifying mathematical theory of pharmacodynamics, that contrasts different antiviral agents according to potency, likelihood of resistance, and therapeutic synergy has only recently been developed [20]–[25], and exists only for HIV-1 targeting agents. To maximize the probability that viral inactivation can be achieved, we believe that key quantitative components of gene therapy should be established early during development of DNA cleavage enzyme technology. With these fundamentals in place, dosing regimens can be designed rationally rather than blindly.

To this end, we developed theoretical models that capture different critical components of viral cure approaches with DNA cleavage enzymes. Our initial models and analyses focus on HBV infection. HBV infects hepatocytes, which are highly accessible to gene therapy delivery vectors and which can be assessed serially for clearance of non-replicating virus. For this reason, HBV may be the most promising initial target for cure. However, the model is easily expanded to account for parameters that govern HIV-1 and HSV infections.

We describe the mathematics of viral vector delivery to hepatocytes, and enzyme - substrate kinetics in the setting of heterogeneous density of episomal infection per hepatocyte. The theoretical problem of *de novo* resistance to DNA cleavage enzyme is also addressed. To this end, we consider concurrent vectorization of multiple enzymes that target separate DNA regions within the HBV episome. Finally, we incorporate simple differential equation models that capture dynamics of HBV persistence between gene therapy doses, and estimate how these dynamics may impact dosing strategies.

Our simulations suggest that therapeutic outcome is likely to hinge on four key factors: percent vector delivery to target cells per dose (which in turn depends on what proportion of vectors are removed by humoral immune mechanisms), enzyme-DNA target binding affinity and cleavage efficiency, degree of binding cooperativity between cleavage enzymes and target DNA, and number of transgenes delivered per vector. We predict that re-accumulation of the latent pool of HBV is unlikely to occur rapidly enough to overcome weekly dosing of delivery vectors, provided that viral replication is concurrently suppressed with available antiviral therapy. If cleavage enzymes that target single regions within the viral genome are used, *de novo* enzyme resistance could develop rapidly such that nearly all remaining episomes are therapy resistant following only a few doses of effective therapy. However, resistance to cleavage enzymes can be effectively mitigated if different DNA cleavage enzymes that cleave different regions of HBV episomes are dosed sequentially, or if single vectors can concurrently deliver several of these transgenes. As this model has yet to be confronted with empirical data, we also discuss potential cell culture and animal model experiments to help identify values for key model parameters, and to better inform future iterations of the model.

## Results

### Key features of persistent HBV infection

The HBV genome can exist in various states within a cell according to stage of replication. The persistent viral form is covalently closed circular (cccDNA), which is maintained with a half-life of months to years in cells [26], but is also a fundamental intermediate in the HBV replication cycle [27]. HBV is notable for an extraordinarily high burden of infection with most of the ∼2×10^11^ human hepatocytes harboring multiple HBV cccDNA episomes [28], as well as other replication intermediates including HBV that may be integrated into host chromosomal DNA [29], [30]. If fully suppressive antiviral therapy is given, the balance of remaining viral molecules is shifted in favor of cccDNA which remains in >95% of cells even a year after HBV DNA becomes undetectable in serum [31]. In model simulations, unless otherwise stated we assume that 99.67% of 2*10^11^ hepatocytes are infected based on a median of five infectious genomes per cell [32], [33]. We replicate the wide distribution of viral burden between cells for HBV with a Poisson distribution. The goal of gene therapy-delivered DNA viral cleavage enzymes will be to functionally disrupt all, or the vast majority of cccDNA, such that viral reactivation is impossible.

While we believe that parameters of gene therapy vector delivery and intracellular pharmacodynamics described in our models can ultimately be precisely identified, the parameter values that govern dynamics of HBV cccDNA persistence are likely to remain undetermined when our therapies are tested in animal and human trials. With these uncertainties in mind, the goal of our models is not to prove or disprove competing dynamical hypotheses of HBV cccDNA persistence [34], but rather to incorporate any possible features of reservoir maintenance that may challenge the effectiveness of gene therapy. As a practical matter, our assumptions are weighted to favor HBV persistence during treatment. We favor “pessimistic” models of latency to ensure that selected gene therapy regimens exceed thresholds for viral cure by a comfortable margin. Because it is generally agreed that cccDNA probably decays slowly even without eradicative therapies, we make the simplest pessimistic assumption, that cccDNA levels remain stable between doses, unless otherwise noted.

### Mathematical model for vector delivery during gene therapy

Gene therapy vectors can be delivered intravenously, allowing random dispersion to target hepatocytes. Entry into target cells can be achieved by utilizing vectors that are engineered to preferentially bind chosen cell surface receptors, such as sodium taurocholate cotransporting polypeptide, which are specific to HBV target cells [35]. Alternatively, viral vectors, which naturally target cell surface receptors that are ubiquitously expressed on the cell surface of target hepatocytes, such as the laminin receptor, heparan sulfate proteoglycans (HSPGs), sialic acids and other glycans, can be used [36]–[41]. During a single dose, all hepatocytes are equally susceptible but delivery of multiple vectors to one cell may occur allowing for multiple transductions. We make the assumption that entry of multiple vectors is not impaired following prior entry of a single vector.

In addition, the model is structured such that gene product, or DNA cleavage enzyme concentration in the cell nucleus is assumed to be directly proportional to the number of delivery vectors with successful entry and gene expression. Recombinant adeno-associated virus (AAV) can be produced in high titers in plasmid transfected cells [42] although non-infectious capsids exceed infectious DNA containing particles 10–30 fold [43]. A total of 60 copies of the capsid protein VP3 are needed to produce a single infectious particle during wild type AAV infections [44], suggesting that generation of a single double-stranded replicating form of AAV vector DNA can correspond with amplified transgene expression.

**Figure 1:**
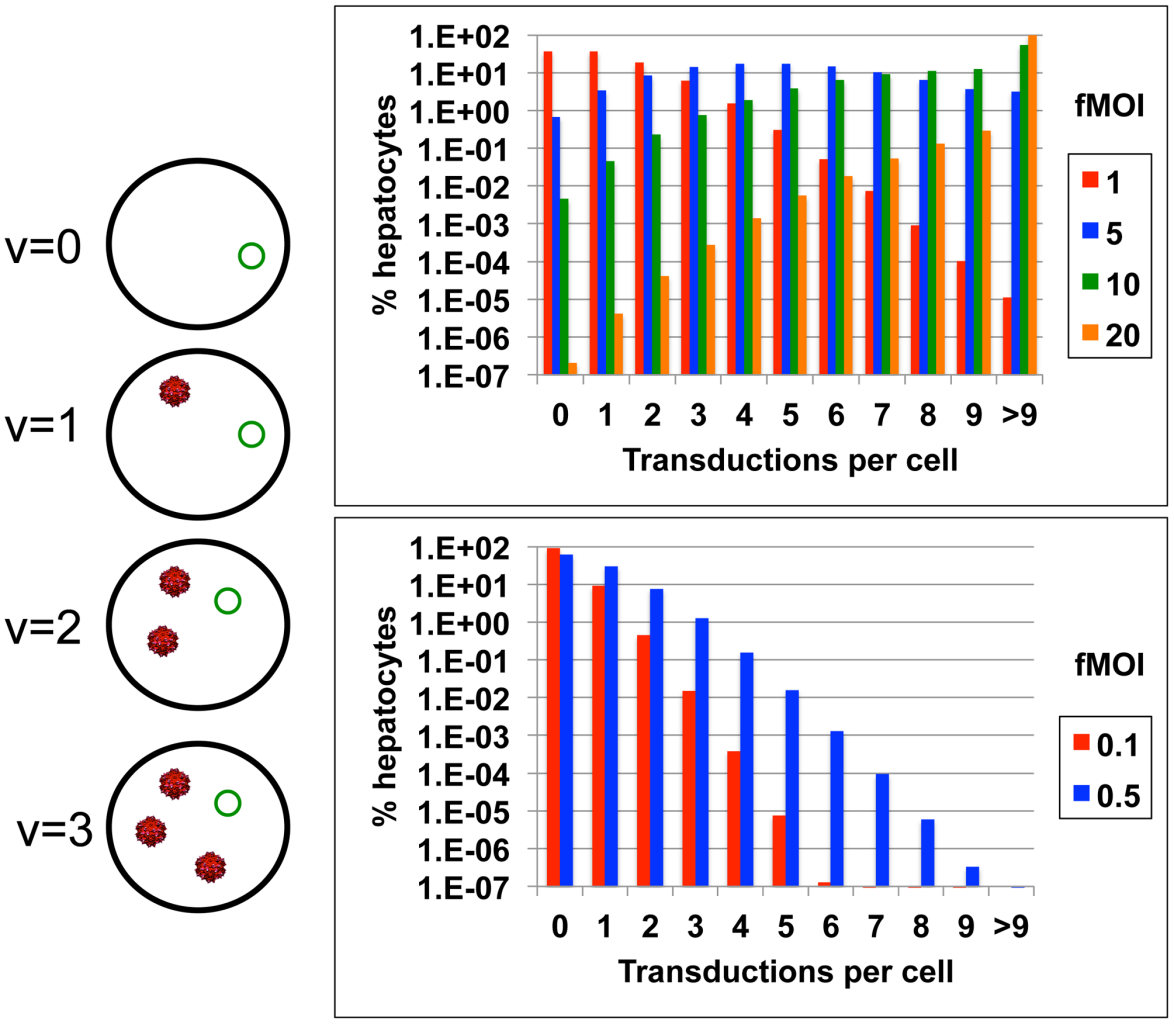
Schematic of gene therapy vector delivery. (a) There is a probability, P_v_ = [(σ*m)^v^ * e^−(σ*m)^]/v!, of different amounts of vector (red) being delivered to and transduced within each cell containing the target virus (green). (b & c) The percentage of cells with different amounts of transduction will vary according to functional multiplicity of infection (fMOI) which is equal to the ratio of transduced delivery vectors to target hepatocytes multiplied by the proportion of vectors which are transduced, or fMOI = m * σ.

The heterogeneous distribution of gene therapy vectors to cells can be captured with an adjusted multiplicity of infection formula P_v_ = [(σ*m)^v^ * e^−(σ*m)^]/v!, where m is the ratio of delivery vectors to target cells in the human body (including uninfected hepatocytes which presumably also take a high number of viral vectors), v is the number of vectors delivered per target cell, and P_v_ is probability of v transduced vectors per cell (**Fig. 1a**). Viral vectors such as AAV have been developed for delivery to the human liver and have successfully deployed at high does (2*10^12^ particles per kilogram) with success in human clinical trials for metabolic disorders [45]. This dose equates roughly to m = 1200, or 1200 particles per hepatocyte in a 60 kg adult.

Parameter σ is included to account for the fact that most vectors will not transduce their intended target cells. As a function of the development process, >90% of vector capsids lack viral DNA [43]. Other vectors may be removed by humoral immune mechanisms, enter cells which are not targets for HBV infection, or degrade due to shear forces or chemical stress in the blood. Finally, vector entry into a target cell's cytosol does not guarantee successful transduction, as viral nuclear localization sequences are required to bind nuclear transport receptors for nuclear entry [46]. Therefore the ratio of transduced vectors per target cells (σ*m), which we refer to as the functional MOI (fMOI), is likely to be far lower than the value for m which is ∼1200.

σ will take on a value of one if transduction of all dosed vectors occurs, and zero if no gene expression is achieved. This parameter value may be lower for infections such as HIV where latently infected cells potentially exist in anatomic sanctuaries such as the nervous system, as compared to HBV where vectors encounter the liver during first pass metabolism. Certain delivery vectors such as adenovirus (ADV) are immunogenic and delivery of identical serotypes will decrease with successive doses [47]. Enhanced neutralizing antibody response can prevent efficient delivery, thereby decreasing σ with successive doses, even when using less immunogenic vectors such AAV [48]–[54].

The delivery equation reveals a wide distribution of vector delivery and transduction when σ*m >1. If HBV infection is modeled with 2*10^11^ hepatocytes, even if 10^12^ vectors are delivered successfully (m = 1200, σ = 0.004, σ*m = 5), there is no transduction within a small percentage (**Fig. 1b**), but relatively large absolute number (∼10^9^), of infected cells. If σ = 0.167 (σ*m = 20) is assumed, then a majority of hepatocytes will have multiple vector delivery (**Fig. 1b**). When σ*m<1, σ*m approximates proportion of cells with delivery and the majority of targeted cells contain only a single delivery vector (**Fig. 1c**).

The latter condition is unlikely to promote complete eradication of HBV cccDNA: if we make the simplifying and overly optimistic assumptions that delivery of one or more vectors automatically leads to lethal mutation of all viral genomes within a target cell, that no immunity to the viral vector or enzyme develops with successive doses of delivery vectors, and that there is no replenishment of infected hepatocytes or HBV cccDNA between doses, then the number of doses prior to eradication can be estimated with the formula N_n_ = N_0_ * (1−P(v>0))^n^ where N_0_ is initial number of infected cells, N_n_ is the remaining number of infected cells following n doses, and cure occurs when N_n_<1. The number of necessary doses increases dramatically if 50% delivery is not achieved while delivery greater than 99% dramatically decreases number of doses needed for cure (**Table 1**). In this analysis, we include HIV and HSV, which have lower numbers of total body latently infected cells (high estimates are 10^7^ and 10^6^, respectively) [55], [56], to highlight that large infectious burden necessitates considerably more doses for elimination of HBV than HIV or HSV (**Table 1**).

**Table 1:** Number of doses prior to viral cure.

Delivery to target cells	Effective MOI (σ * m)	HBV	HIV	HSV
**99.9999%**	13.8	2	2	1
**99.999%**	11.5	3	2	2
**99.99%**	9.2	3	2	2
**99.9%**	6.9	4	3	2
**99%**	4.6	6	4	4
**95%**	3.0	9	6	5
**90%**	2.3	12	8	7
**75%**	1.4	19	14	12
**50%**	0.69	38	26	22
**25%**	0.29	91	59	50
**10%**	0.10	247	154	132

Here we assume that successful delivery and gene transduction automatically leads to inactivation of all viral genomes within an infected cell.

This analysis highlights the importance of high vector to target cell ratio, even under favorable assumptions regarding intracellular pharmacodynamics. Because the value of parameter m will be known as a function of dose, the key unknown parameter of delivery is σ, the proportion of vectors that enter target cells and are transduced.

### Basic intracellular pharmacodynamics of DNA cleavage therapy

Two factors will drive outcome of an infected cell following delivery of transgene-carrying vectors: the number of viral vectors transduced in the cell and the strength of the enzyme-substrate interaction. The critical biophysical interactions are the binding affinity between enzyme and substrate, the efficiency of enzyme cleaving following binding and the efficiency of precise DNA repair. These processes are captured indirectly with constant d in the formula λ_o_ = 1/(1+(v/d)) where v is number of vectors transduced in the cell and λ_o_ is probability that the genome will remain uncleaved. In this formula, d is scaled according to vector gene expression value per cell under the assumption that intracellular enzyme concentration is directly proportional to v [44]. The value of d determines whether one or multiple vectors will need to be delivered to the nucleus to ensure terminal mutation of most viral episomes. If d<<1, then transduction of one vector is likely to predict episomal cleavage. Alternatively, if d>1, then multiple vectors per nucleus will be necessary to disrupt all viral DNA.

### Resistance to DNA cleavage enzymes

A possible hurdle to disruption of latent genomes is resistance to the cleavage enzyme in question. cccDNA molecules may contain pre-existing mutations. The HBV mutation rate is relatively low [57], and pre-existing mutations to cleavage enzymes are likely to be relatively rare. DNA cleavage enzymes may also induce *de novo* mutations that render the site resistant to subsequent enzyme binding but do not incapacitate the virus. For example, if an enzyme repair event results in a 3 base pair mutation within the open reading frame, the ensuing loss of a single amino acid may theoretically not impair activity of the viral protein. However, if the DNA cleavage site is no longer recognized by the cleavage enzyme then this site has effectively become “enzyme resistant”. Presumably, this process will occur at a relatively low rate. For each cleavage and mutation event, the maximum probability of resistance is 33% as a deletion or insertion with a multiple of 3 is a pre-requisite for this event. However, addition or removal of one or several amino acids from the viral gene product will prove fatal to the virus on most occasions. Based on preliminary data using a target site in the N-terminus of a green fluorescent protein in a non-functional region, an absolute upper possible estimate is that ∼5% of cleavage/mutations events may result in *de novo* resistance [11], though we expect the actual rate to be considerably lower. If probability of cleavage is P_c_ = (1−λ_o_), then the probability of resistance is P_r_ = P_c_ * Ψ where Ψ is the frequency of induced mutations that prevent further enzyme binding despite being non-lethal to the viral episome. Therefore, in our model, development of resistance is assumed to increase proportionally with amount of DNA cleavage. To isolate the more important effects of induced *de novo* mutations, we include no pre-existing mutations in model simulations.

### Possible therapeutic outcomes with multiple viral episomes per cell

**Figure 2:**
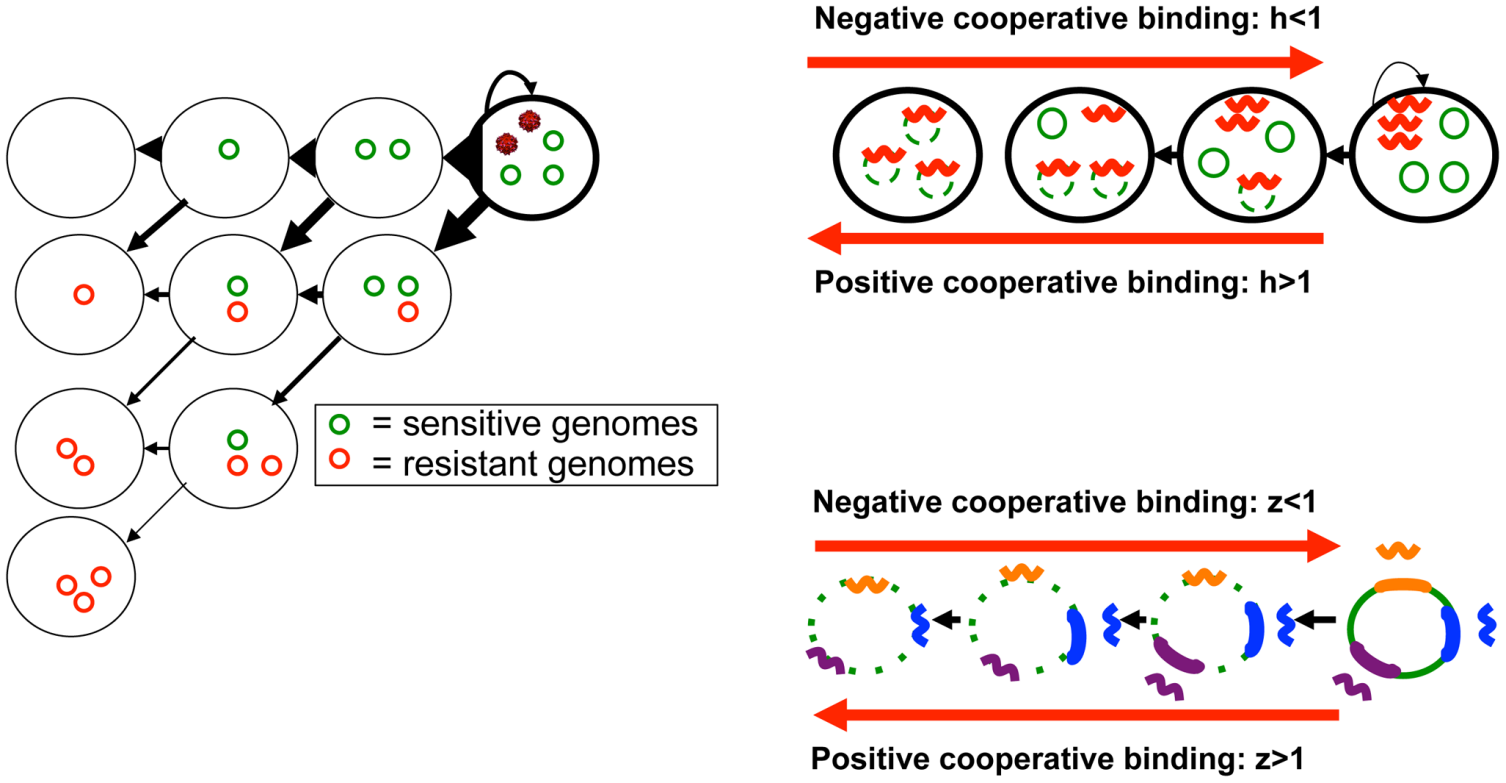
Intracellular HBV DNA cleavage enzyme pharmacodynamics. (a) An HBV infected cell with three cccDNA molecules (green circles), and delivery of DNA cleavage enzyme containing vectors (red viruses) can transition to several states where none, some, or all of the episomes are eliminated and/or become resistant to the cleavage enzyme. Arrow thickness denotes the relative probability of each event. (b) Cleavage enzymes (red wavy lines) may bind HBV cccDNA molecules cooperatively, whereby binding of one enzyme to its target sequence enhances binding of other enzymes to the same target on separate episomes. (c) Cleavage enzymes (multi-colored wavy lines) that target separate regions within episomes (thick colored lines of corresponding color) may bind HBV cccDNA molecules cooperatively, whereby binding of one enzyme to its target sequence enhances binding of other enzymes to separate sequences on the same episome.

Most persistent HBV exists as multiple non-replicating episomes within infected cells. For this reason, outcomes for a cell with intra-nuclear cleavage enzyme expression include only partial inactivation of genomes, as well as development of *de novo* resistance to cleavage enzymes in some but not all remaining viral molecules. The number of possible transition states of an infected cell following delivery of a vector is a function of the number of genomes within the cell (**Fig. 2a**): all or a portion of episomes can be disrupted by DNA cleavage, while all or a portion of disrupted episomes can develop *de novo* resistance. Each transition state has a certain probability following delivery of a certain number of delivery vectors, including the probability that the infected hepatocyte will undergo no change in its state. In general, development of resistance is less common than successful disruption and elimination of viruses (**Fig. 2a**). The total number of cells undergoing each transition is estimated by multiplying individual transition probabilities, by the number of cells with a certain number of cccDNA molecules, and amount of vector delivered.

### Enzyme-substrate binding cooperativity

Enhanced cooperative binding between HIV directed antiviral agents and their multivalent viral enzyme targets has been demonstrated as a key determinant in antiviral agent potency. For example at equivalent drug concentrations, HIV protease inhibitors can be 100,000 times more potent than HIV nucleoside reverse transcriptase inhibitors [20]. Similarly, enzyme binding to a single viral episome may enhance or impair binding of subsequent enzymes to neighboring episomes in the nucleus (**Fig. 2b**). Moreover, if multiple enzymes that target distinct genomic regions within a single cccDNA episome are dosed simultaneously, then there may be enhanced or impaired binding to these multiple episomal sites (**Fig. 2c**).

The mechanism to determine whether cooperative binding is present is generation of log-converted dose response curves with a particular emphasis on the slope of the curve, which translates to Hill coefficient (h*z) in the formula λ_o_ = 1/(1+(v/d)^h*z^). Parameter h represents enhanced binding of one enzyme product to multiple intranuclear episomes (**Fig. 2b**). A value of parameter h greater than one implies positive cooperative binding and will favor cleavage of multiple episomes (**Fig. 2b**), while a value less than one implies binding competition and will favor cleavage of only a single episome per transduction event. Under extreme conditions of negative binding cooperativity, the number of gene therapy doses will need to be equivalent to the maximum number of genomes per cell.

**Figure 3:**
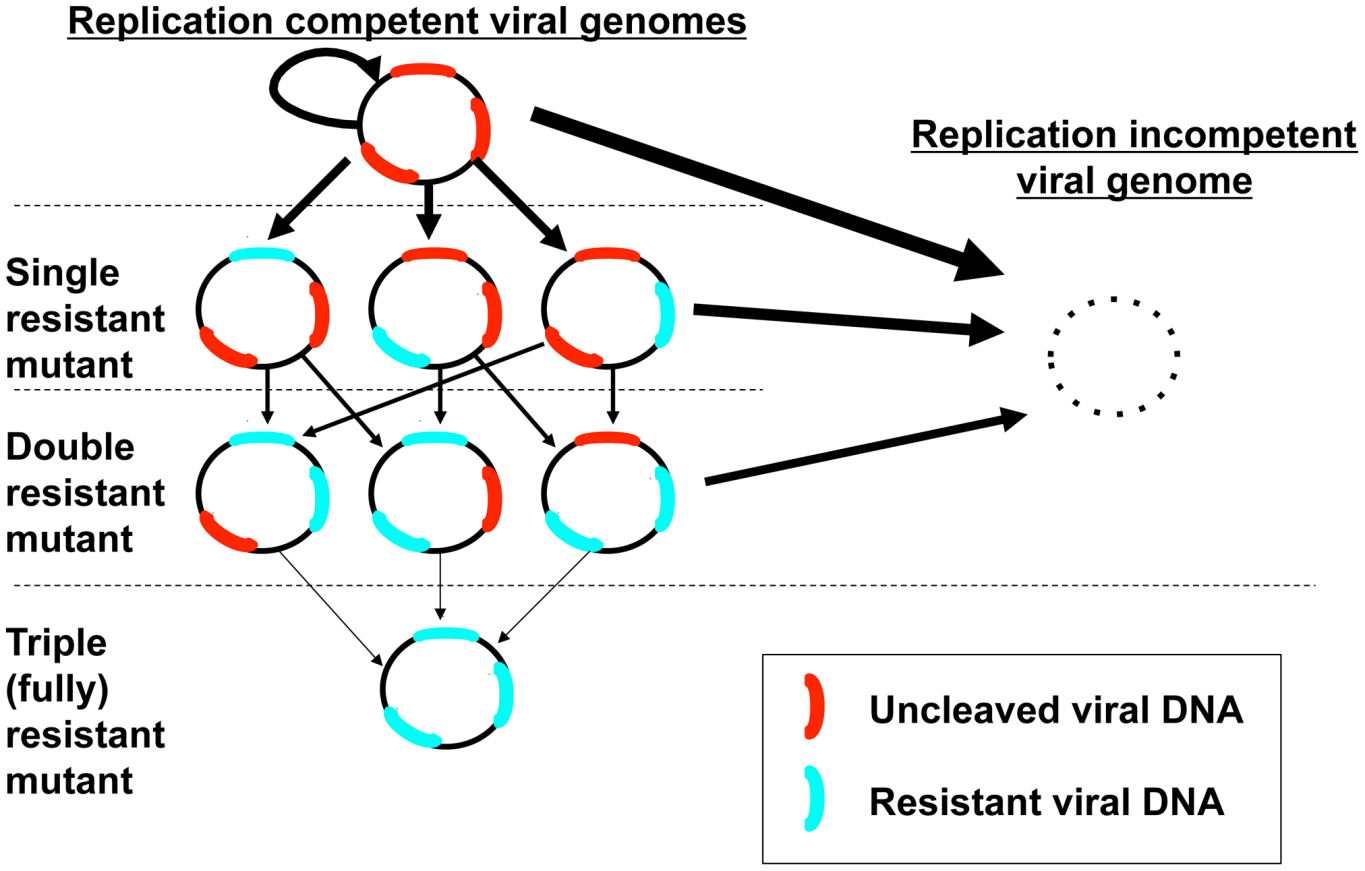
Possible outcomes for a single episome with three possible cleavage sites. Any cleavage event that renders the molecule replication incompetent is a terminal event for the episome; induced resistance at one site still leaves other potential target sites susceptible to cleavage. Arrow thickness denotes the relative probability of a certain event.

Parameter z represents the possibility of enhanced or impaired binding of multiple enzymes products to one viral genome at separate binding sites (**Fig. 2c**). If only one episomal DNA sequence is targeted, then z = 1 and the Hill coefficient is reduced to parameter h alone. The presence of multiple cleavage enzyme targets may be necessary to avoid resistance: while only one successful cleavage event will usually be required to neutralize replication activity of the episome, if cleavage at a certain site induces *de novo* resistance, then a different enzyme will need to bind a separate site to terminally disrupt the episome (**Fig. 3**). Under this set of rules, enhanced binding to secondary sites may prove advantageous. An episome that becomes resistant to all available enzyme products and maintains replicative capacity is termed fully resistant (**Fig. 3**). To reflect that parameters h and z may have opposing or complementary effects, they are included as a product in the equation. Also of note, parameter z may take on different values for different enzymes that are concurrently dosed, though for the purpose of theoretical simulations, we assume a single value.

### Transitions in burden of infection following gene therapy infusion

Assuming that potency of a single DNA cleavage enzyme (z = 1) on an individual cccDNA episome level is captured with the equation λ_o_ = 1/(1+(v/d)^h^) where λ_o_ is probability of the episome remaining uncleaved, total cleavage enzyme activity within a single cell is represented by P_c_(i) = (^S^_i_) * (1−λ_o_)^i^ * (λ_o_)^(S−i)^ where a cell has S enzyme susceptible cccDNA genomes and P_c_(i) represents the probability of cleaving *i* episomes. At high levels of v/d, the probability of cleaving all episomes within a cell, or (1−λ_o_)^S^, increases. Resistance to cleavage enzymes occurs as a function of cleavage events. Given *i* cleaved episomes within a cell, *k* episomes will become resistant according to formula: P_r_(k) =  = (^i^_k_) (Ψ)^k^(1−Ψ)^(i−k)^.

To synthesize these concepts for HBV infection, we created a three-dimensional matrix. This model tracks total number of cells occupying different states over time. Between cleavage enzyme doses, the numbers of cells with every possible combination of replication competent enzyme susceptible (S) and enzyme resistant (R) genomes are measured. A third dimension is incorporated following each infusion of therapy, and accounts for different doses of vector transduction: each item within the matrix represents the total number of infected cells with a certain value for S, R and v. Transition probabilities are calculated for each cell according to P_c_(i) and P_r_(k). The matrix is updated accordingly following each dose (**Fig. 2a**).

Initial data suggest that enzyme activity and DNA mutations accrue over a week following vector delivery [11]. In practice, delayed enzyme activity following vector entry into target cells would prove problematic only if cccDNA levels reconstitute in a meaningful way during the time period between doses. Otherwise, dosing interval can simply be prolonged to wait for enzymes to exert their full effect, and this would not impair the probability of therapeutic efficacy. In the simulation model, for simplification purposes, DNA cleavage is assumed to occur instantly following delivery of vectors with a dosing interval of one week. In later model realizations, the possible effects of cccDNA reconstitution and slower enzyme onset are explored.

### Sequential delivery of multiple DNA cleavage enzymes with single enzymes per vector to avoid resistance

**Figure 4:**
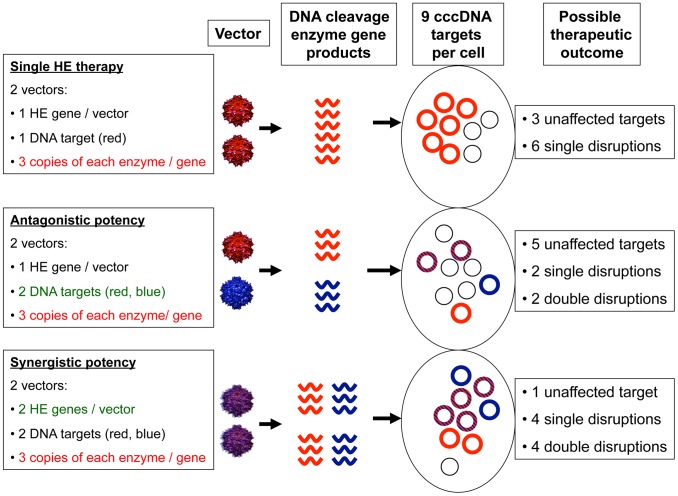
Theoretical effects of dosing multiple enzymes concurrently. If two enzymes targeting separate sites are split among vectors, then “antagonistic potency” may occur: while the likelihood of *de novo* resistance decreases because fewer episomes are cleaved at only a single site, fewer episomes are targeted overall. “Synergistic potency” is more likely to occur if two enzymes are packaged within the same vector: intracellular dose of enzyme will double leading to fewer unbound episomes and lower probability of resistance due to higher overall binding of episomes at two sites. The possible therapeutic outcomes illustrated in the diagram are one of hundreds of potential outcomes given nine pre-therapy cccDNA molecules per cell, and are intended only as a demonstration of this principle.

Strategies to bypass enzyme resistance will be analogous to those employed for antiviral therapy, namely design of cleavage enzymes that target separate regions within episomal HBV cccDNA (**Fig. 2c** **,** **3**). Several possible dosing schemes exist (**Table 2**). Smaller vectors such as AAV can probably only carry 1–2 open reading frames, though different serotypes can theoretically be given with each successive dose with the goal of avoiding a strong humoral immune response. If AAV is employed, then multiple enzymes that target separate sites must be divided between separate vectors. These vectors can be dosed concurrently (thereby decreasing the delivery dose of each vector/enzyme combination). While this strategy will theoretically increase the proportion of genomes targeted with two enzymes, the overall number of eradicated genomes may decrease due to overlapping targeting within the genome leading to a lower overall number of targeted episomes: we term this hypothetical problem “antagonistic potency” (**Fig. 4**).

**Table 2:** Dosing schemes.

	One enzyme/vector, single enzyme (q = 1)	One enzyme/vector, multiple enzymes (q≥1), sequential delivery^1^	Multiple enzymes (q≥1)/vector^2^
**Delivery equation**	P_v_ = [(σ*m)^v^ * e^−(σ*m)^]/v!	P_v_ = [(σ*m)^v^ * e^−(σ*m)^]/v!	P_v_ = [(σ*m)^v^ * e^−(σ*m)^]/v!
**Single episomal cleavage equations**	λ_0_ = 1/(1+(v/d)^h^)	λ_0_ = 1/(1+(v/d)^h^)	λ_0_ = 1/(1+(v/d)^h*z^)
**Multiple episomes cleaved per cell equations**	P_c_(i) = (^S^_i_) (1−λ_0_)^i^(λ_0_)^(S−i)^	1^st^ dose: P_c_(i) = (^S^_i_)(1−λ_0_)^i^(λ_0_)^(S−i)^ Subsequent doses: P_c_(i) = (^Stot^_i_)(1−λ_0_)^i^(λ_0_)^(Stot−i)^	1^st^ enzyme: P_c_(i) = (^S^_i_)(1−λ_0_)^i^(λ_0_)^(S−i)^ Subsequent enzymes: P_c_(i) = (^Stot^_i_)(1−λ_0_)^i^(λ_0_)^(Stot−i)^
**Resistance equations**	P_r_(k) = (^i^_k_) (Ψ)^k^(1−Ψ)^(i−k)^	P_r_(k) = (^i^_k_) (Ψ)^k^(1−Ψ)^(i−k)^	P_r_(k) = (^i^_k_) (Ψ)^k^(1−Ψ)^(i−k)^

1 = Delivery occurs between each of q successive enzyme exposure.

2 = Delivery accounts for cellular levels of each of q multiple co-packaged enzymes.

q = number of cleavage enzymes, number of viral sites targeted by cleavage enzymes.

S = number of fully susceptible episomes per cell.

R_1….q_ = number of episomes resistant to 1…q cleavage enzymes per cell.

v = # vectors delivered per cell.

m = vector dose/# of target cells.

σ = proportion of vectors that reach uninfected or infected target cells following delivery.

λ_0_ = probability of a single episome remaining uncleaved.

d = binding coefficient.

h = “between episome” Hill coefficient for a single enzyme.

z = “within episome” Hill coefficient for multiple enzymes.

P_c_(i) = probability that *i* episomes are cleaved in a cell with S susceptible episomes.

S_tot_ = S+R_1_+R_2_+R_q−1_.

Ψ = resistance rate per cleavage event.

P_r_(k) = probability that *k* episomes develop *de novo* resistance given cleavage at *i* episomes within a cell.

While a very high effective fMOI (>10 in **Table 1**) may overcome antagonistic potency, another approach would be to dose separate cleavage enzymes within AAV successively rather than concurrently. With sequential delivery of enzymes targeting different regions, the vector delivery equation remains unchanged. The equation λ_0_ = 1/(1+(v/d)^h^) again describes the probability of a genome remaining uncleaved. If q enzymes are available, then all remaining replication competent episomes are assumed to remain sensitive to subsequent doses through the first q doses (assuming a different enzyme is used with each dose). In other words, resistance to the first delivered enzyme will not impact activity of the second enzyme and so on. Transitions are mediated by P_c_(i) = (^Stot^_i_)(1−λ_0_)^i^(λ_0_)^(Stot−i)^, where S_tot_ is the number of total episomes in a cell (either susceptible or resistant to prior delivered enzymes). Enzyme resistance is again captured with P_r_(k) = (^i^_k_) (Ψ)^k^(1−Ψ)^(i−k)^ and generation of single and multiple mutants is tracked following each dose.

**Figure 5:**
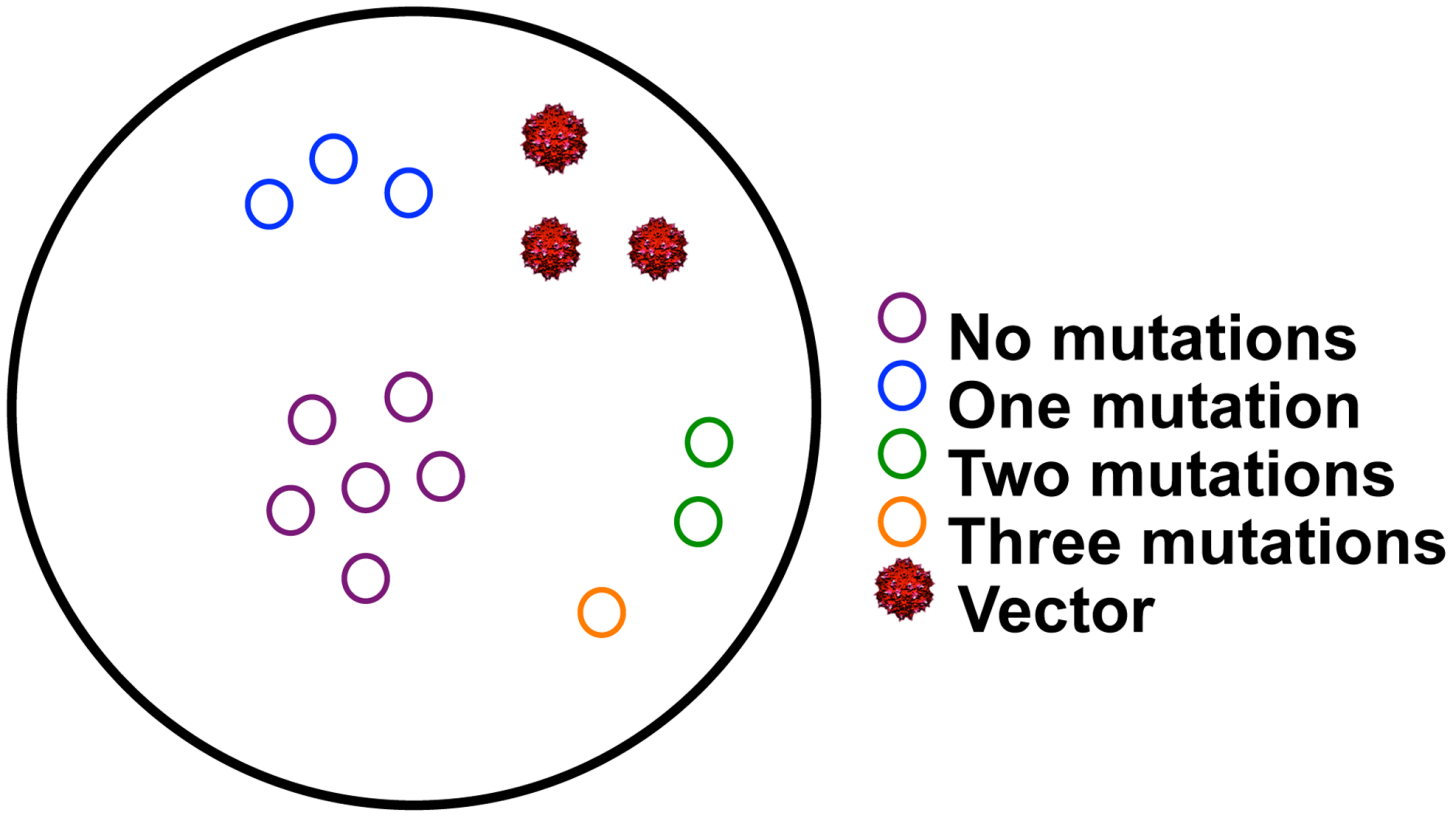
A single cell may contain episomes with different degrees of resistance to multiple enzymes. Example of one theoretical cell containing 6, 3, 2 and 1 episomes with 0, 1, 2, and 3 mutations respectively, as well as 3 delivery vectors.

Each cell within the liver may harbor different numbers of episomes with zero, single and multiple resistant sites (**Fig. 5**). We add a new dimension to the matrix with each delivery of a new cleavage enzyme such that the matrix contains q+2 dimensions given q total enzymes. For instance, a simulation with q = 3 (3 sequentially dosed enzymes with different DNA target sequences) will include the following dimensions: S (non-resistant episomes), R1 (single resistant episomes), R2 (double resistant episomes), R3 (triple of fully resistant episomes) and v (vectors). Transitions to a newly resistant state are mediated by prior resistant state of the episome: with development of *de novo* resistance, S transitions to R1, R1 transitions to R2, and R2 transitions to R3. S_tot_, defined above, is the sum of S, R1 and R2.

The model output is constructed in one of two ways: either the number of episomes with any resistance (S_tot_−S) are plotted against number of fully susceptible episomes (S); or the number of episomes with total resistance to all episomes (SR_tot_) are plotted against number of remaining episomes without total resistance (S_tot_−SR_tot_). Only after q doses are given is it possible to have SR_tot_>0 due to totally resistant episomes to each of the q available enzymes. Following q doses, our model assumes repeated dosing of the finally dosed enzyme, as the least number of resistant episomes will exist against this enzyme.

### Multiple, concurrent DNA enzyme delivery per vector to avoid resistance

If vectors such as ADV with higher gene payload capacities are utilized, then two or more separate enzymes can be delivered and transduced within the same vector. This approach has the theoretical advantage of increasing per cell dose of cleavage enzyme, and has the potential to increase the proportion of targets that receive multiple cleavage enzymes, a process we term “synergistic potency” (**Fig. 4**). Moreover, if z>1 due to enhanced binding cooperativity between enzymes (**Fig. 2c**), then cccDNA cleavage will be augmented in this fashion as well. Unfortunately, ADV is highly immunogenic and may only achieve high delivery following the first dose, and would need to be followed with AAV or other smaller delivery vectors, or ADV of different serotypes.

Our model allows for analysis of potential benefits gained from delivery of multiple transgenes within a single ADV vector. The delivery equation is unchanged from prior simulations, as the number of vectors and therefore proportion of cells with no vector transduction (P_v_ = 0) remain the same. If a vector carries *q* enzymes, then intracellular concentration of cleavage enzyme increases by a factor q (**Fig. 4**). We isolate the compounded effects of multiple enzymes, as well as the possible accrual of multiple enzyme resistant mutants by sequentially evaluating the activity of individual enzymes within a cell using P_c_(i) = (^Stot^_i_)(1−λ_0_)^i^(λ_0_)^(Stot−i)^, where S_tot_ is again equal to the number of total episomes in a cell (either susceptible or resistant to prior evaluated enzymes). Resistance is captured with P_r_(k) = (^i^_k_) (Ψ)^k^(1−Ψ)^(i−k)^ and generation of single and multiple mutants is tracked following each dose. The matrix again contains q+2 dimensions. The critical difference between sequential dosing and multiple enzyme delivery simulations is that for the latter, delivery is not updated between successive evaluation of enzyme activity. Only after all of the q enzymes are evaluated, do we sum the number of totally resistant (SR_tot_), partially resistant (S_tot_−SR_tot_−S) and susceptible (S) episomes in liver cells to update infectious burden within the entire liver.

### HBV therapeutic cure simulations assuming no *de novo* resistance

To demonstrate characteristics of the model, we conducted simulations under different assumptions of vector delivery (fMOI), enzyme-substrate binding avidity/cleavage efficiency (binding dissociation constant or d), and cooperative binding of enzymes to multiple episomes (Hill coefficient or h). Initial simulations assumed a single transgene per vector and ignored *de novo* resistance. Pre-therapy conditions assumed fully suppressive antiviral therapy, a median of 5 episomes per cell, no inherent decay of infected cells or HBV cccDNA over time, and a total of 10 weekly doses. We defined infected cells as any cell with at least one remaining replication competent HBV cccDNA molecule. In initial simulations, we also assumed that the effect of each dose occurred instantaneously.

First, we performed a multi-parameter sensitivity analysis with parameter values drawn randomly from a pre-determined wide range (fMOI 0.5–5, binding dissociation constant 0.008–5, and Hill coefficient 0.2–5) using Monte Carlo selection methods. We generated 200 parameter sets and simulated the model to identify parameter effects on therapeutic outcome. Increasing fMOI (R^2^ = 0.50), and decreasing binding dissociation constant (R^2^ = 0.24) predicted lower remaining numbers of infected cells to a greater extent than increasing the Hill coefficient (R^2^ = 0.03).

**Figure 6:**
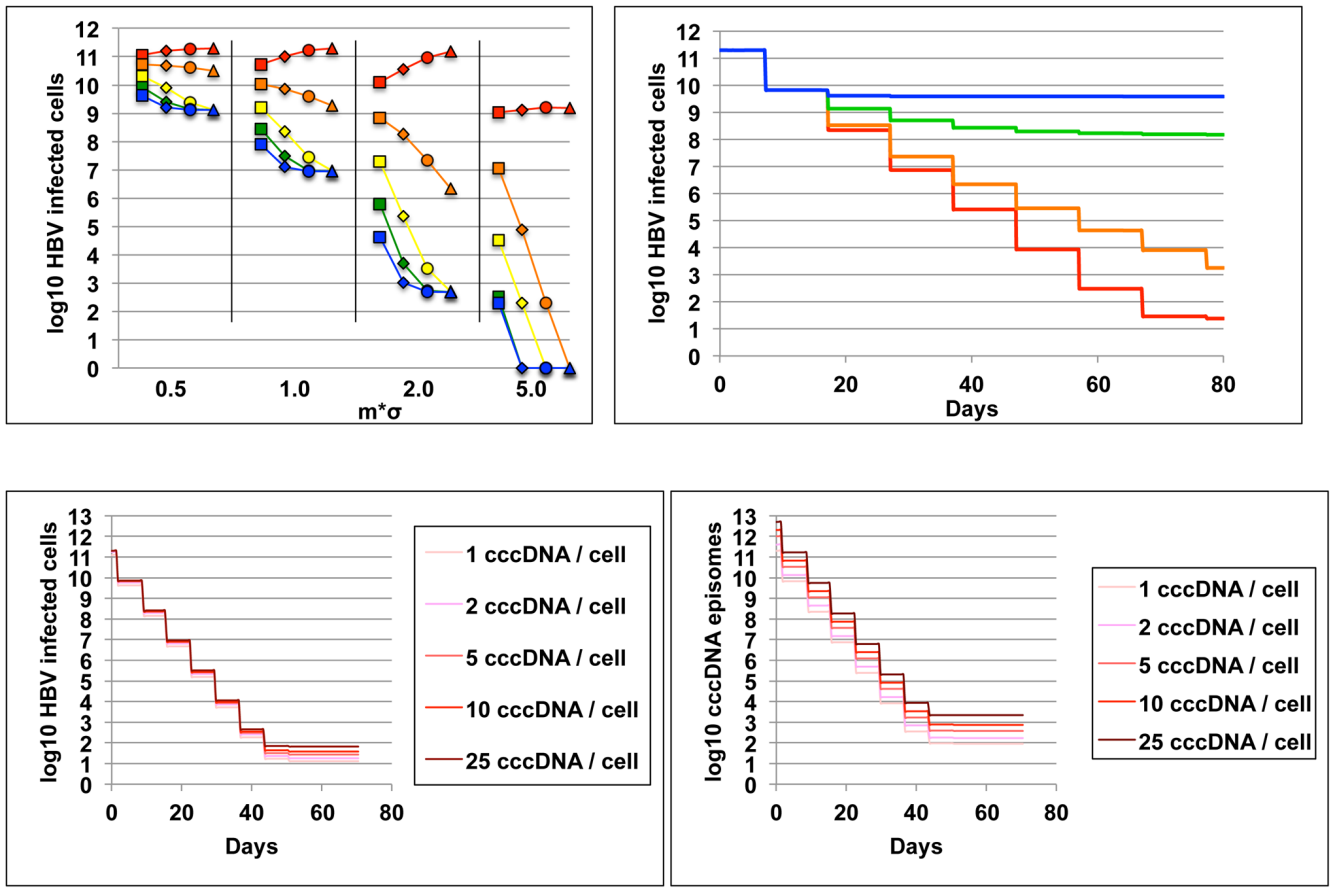
High vector delivery, effective enzyme-HBV binding and cooperative binding predict effective cccDNA clearance. All simulations of HBV eradication show results of ten weekly doses of therapy. A single enzyme is used and *de novo* resistance is ignored. (a) Each data point represents number of remaining infected cells (y-axis) after a simulation with one of 80 unique parameter sets. x-axis is functional multiplicity of infection (fMOI, separated by vertical black lines and not according to scale). Five different values for enzyme-DNA binding dissociation constant (d = 0.008, 0.04, 0.2, 1 & 5) are represented by blue, green, yellow, orange & red respectively; squares, diamonds, circles and triangles represent different values for the Hill coefficient (h = 0.5, 1, 2, 5). High fMOI, low binding dissociation constant and under conditions of moderate delivery and enzyme-DNA binding, high Hill coefficient (cooperative binding), predict high therapeutic potency. (b) Simulations of 10 weekly doses of a potent regimen (fMOI = 5.0, d = 0.04, h = 2, *de novo* resistance rate (Ψ) = 0) with decreasing fMOI following each dose due to humoral immunity. (σ: blue, green, orange and red represent decreases in fMOI with each dose of 90%, 50%, 10% and 0% respectively). Removal of vectors following each dose decreases effectiveness of therapy. (c & d) Simulations of 10 weekly doses of a potent regimen (fMOI = 5.0, d = 0.04, h = 2, *de novo* resistance rate (Ψ) = 0) assuming different burdens of infection (line color represents pre-therapy median number of HBV cccDNA molecules/cell) demonstrate relatively similar potency across highly variable densities of infection whether (c) infected cells or (d) total episomes are tracked as measures of therapeutic outcome.

To obtain a more mechanistic understanding of how model parameters interact to impact the extent of episome disruption, we created 80 parameter sets derived from 4 possible values for fMOI, 4 possible values for the Hill coefficient, and 5 possible values for binding dissociation constant. Model simulations were stochastic but produced equivalent results for repeat experiments with each parameter set. At low fMOI (m*σ = 0.5), decreasing the dissociation constant and/or increasing the Hill coefficient only allowed for a slight relative decrease in number of infected cells following 10 doses; at higher levels of vector delivery, each 5-fold decrease in the dissociation constant (change in color in **Fig. 6a**) resulted in a substantial decrease in infected cells following 10 doses. Increasing the Hill coefficient from 1 to 5 had a similar effect (change in shape in **Fig. 6a**), though this effect was absent at the highest simulated dissociation constants (all red lines in **Fig. 6a**), because a threshold of intracellular enzyme density was not surpassed to allow enhanced cooperative binding. At high fMOI and very low dissociation constants, episome binding saturated with or without the presence of enhanced cooperative binding (blue line under fMOI = 5 in **Fig. 6a**). Residual replication competent genomes during simulations with low dissociation constant and high binding cooperativity resulted from lack of vector delivery to a subset of cells (fMOI = 0.5 or 1.0) rather than lack of enzyme activity within infected cells.

If we assumed that humoral immunity removed an increasing proportion of vectors prior to delivery with each dose (successive decreases in parameter σ), then a greater number of cells retained replication competent episomes following 10 doses even with a potent regimen (**Fig. 6b**).

However, pre-treatment burden of infection as measured by median number of cccDNA episomes per cell prior to initiation of gene therapy, had only a small impact on remaining number of infected cells (**Fig. 6c**) and total replication competent episomes (**Fig. 6d**) following 10 equivalently potent doses of therapy.

### HBV therapeutic cure simulations assuming *de novo* resistance

**Figure 7:**
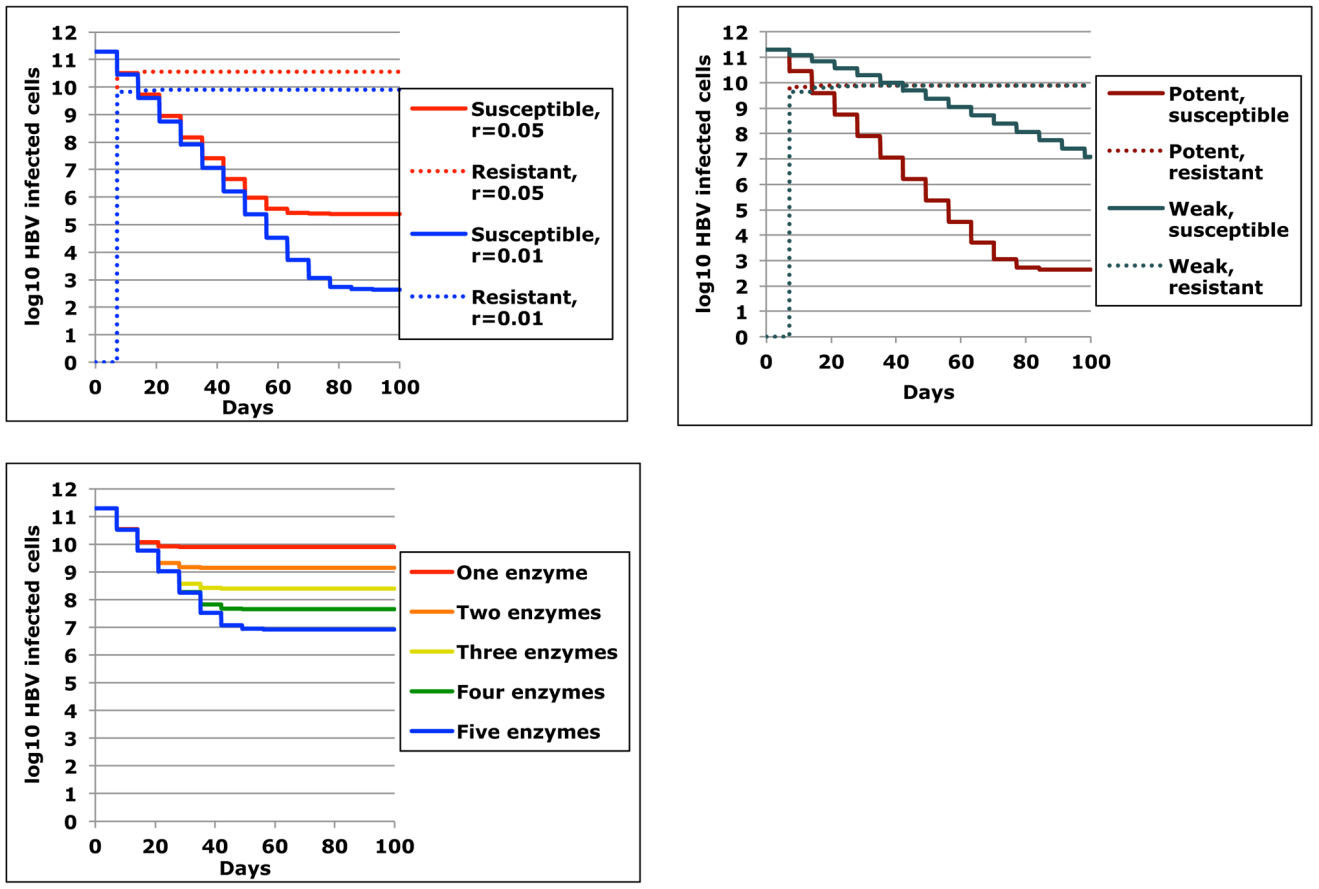
Rapid development of *de novo* resistance to DNA cleavage enzyme therapy. (a) Potent regimens with high fMOI (m*σ = 5), high enzyme – DNA binding avidity (d = 0.04), and positive binding cooperativity (h = 2) will allow for high levels of simulated resistance and predominance of resistant episomes following only 2 to 3 doses; a higher resistance rate (5% versus 1%) will promote a higher number of infected cells containing enzyme resistant episomes. (b) Infected cells containing enzyme resistant episomes will ultimately achieve equivalent levels assuming equal resistant rates whether a potent (m*σ = 5, d = 0.004 & h = 2) or less potent (m*σ = 1, d = 1 & h = 2) regimen is used. (c) If successive enzymes are dosed that target different regions within HBV cccDNA episomes, then the number of remaining episomes following multiple doses decreases accordingly; susceptible and resistant replication competent genomes are summed; by 60 days, all remaining episomes are resistant to each of the dosed enzymes (not shown in diagram).

If *de novo* enzyme resistance developed at a fixed rate per cleavage event and single enzyme therapy was assumed, then resistant genomes rapidly predominated following dosing with parameter combinations that would constitute potent regimens. If we assumed high delivery, avid enzyme – DNA substrate binding and positive binding cooperativity, and that the resistance rate was 5% or 1% per cleavage event, then only 2 or 3 doses were needed respectively prior to infected cells containing resistant genomes becoming the predominate infected cells. In addition, the set point of number of cells with resistant genomes was >0.5 log higher with an assumed resistance rate of 5% versus 1% (**Fig. 7a**). More potent regimens lead to more rapid predominance of cells with resistant HBV cccDNA but if enough doses were given, the set point of number of infected cells with resistant HBV was equivalent between more and less potent regimens with lower fMOI and higher dissociation constant, assuming equal probability of resistance per cleavage event (**Fig. 7b**). With potent regimens and a resistance rate of 5%, cells with multiple HBV episomes harbored a combination of susceptible and resistant forms, though many cells developed multiple resistant episomes, even after a single dose (**Movie S1**). Therefore, a key parameter to deduce experimentally will be rate of resistant mutants generated per cleavage event.

To avoid cleavage enzyme resistance, we next considered sequential delivery of 1, 2, 3, 4, or 5 enzymes in separate, weekly doses. A new enzyme was given each week until no new enzymes remained (at the sixth dose for the 5 enzyme condition, for example). At this point, the final enzyme was repeatedly redosed. Simulations assumed favorable potency parameters and a resistance rate of 1%. The addition of extra enzymes increased the time until enzyme resistant forms predominated, and lowered the steady state of cells retaining replication competent HBV cccDNA by ∼0.5 log with addition of each enzyme (**Fig. 7c**). Simulations with multiple successive enzymes resulted in lower numbers of infected cells than simulations with a single enzyme (dotted lines **Fig. 7a**, red line **Fig. 7c**). Yet, high numbers of enzyme resistant episomes still remained even following sequential dosing of five different enzymes (blue line, **Fig. 7c**).

**Figure 8:**
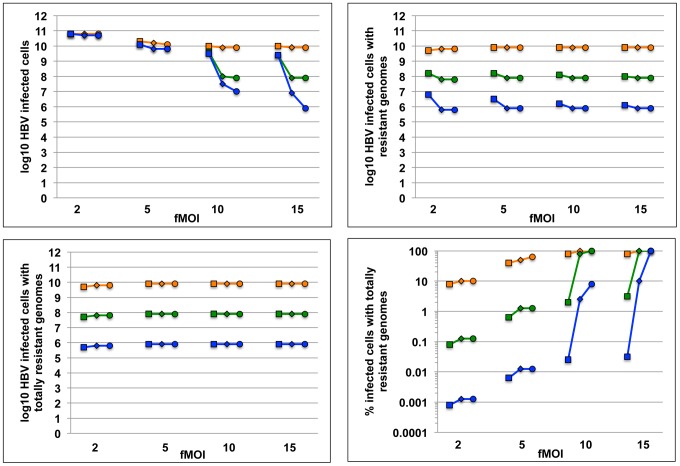
Packaging of multiple cleavage enzymes that target different HBV regions enhances potency and decreases resistance. Simulation of HBV eradication employing gene therapy following a single dose. Each data point represents number of remaining infected cells (y-axis) after a simulation with one of 36 unique parameter sets. x-axis is functional multiplicity of infection (fMOI). Enzyme-DNA binding avidity is fixed (d = 0.04). Color represents number of enzymes delivered per vector (orange, green and blue = 1,2 & 3 respectively). Hill coefficient is 1, 2 and 5 (square, diamond and circle). The simulation assumes no pre-existing resistance. (a) Addition of multiple DNA cleavage enzymes within single vectors decreases the number of total remaining infected cells, particularly when vector delivery is high and intracellular binding cooperativity is present. (b) Addition of multiple DNA cleavage enzymes within single vectors decreases the number of total remaining infected cells harboring HBV cccDNA with any *de novo* resistance mutations, or (c) all possible resistance mutations. (d) Percentage of remaining infected cells containing totally resistant genomes following a single dose increases with high delivery, lower number of cleavage enzymes per vector, and higher binding cooperativity.

We next simulated trials with a single dose of a multi-payload vector such as ADV carrying 1, 2, or 3 transgenes concurrently under different assumptions of fMOI and cooperative binding of enzymes to multiple episomes. A favorable enzyme-substrate binding avidity/cleavage efficiency was assumed for each simulation. Results from simulations with 36 pre-selected parameter sets (all following a single dose with assumed resistance rate = 1%) show that total remaining cccDNA genomes decreased with increasing fMOI, and that maximizing the transgene payload (blue line, **Fig. 8a**) increased effectiveness under high delivery conditions, especially in the presence of positive cooperative binding (circles, **Fig. 8a**), or lower dissociation constant (not shown). Even under lower delivery conditions (fMOI = 2), increasing the number of enzymes per vector dramatically decreased the total burden of infected cells containing viral genomes with at least one *de novo* enzyme resistance mutation (**Fig. 8b**) as well as the total number of infected cells containing HBV cccDNA molecules that were fully resistant to all of the q available delivery enzymes (**Fig. 8c**). Concurrently delivered DNA cleavage enzymes therefore are predicted to exhibit synergistic potency and decrease both the overall burden of infection and *de novo* resistant genomes (**Fig. 4**). Delivery remained a critical parameter for HBV cccDNA disruption and at low fMOI, most remaining cccDNA episomes were susceptible to the cleavage enzymes (**Fig. 8d**). Alternatively, delivery of multiple enzymes generally decreased percent of remaining episomes that were resistant. Positive binding cooperativity between enzymes generally increased the proportion of enzyme resistant episomes by virtue of its overall positive impact on cleavage: a similar effect occurred with lowering the binding dissociation constant (data not shown).

### Therapeutic cure simulations with varying assumptions of HBV cccDNA dynamics

**Figure 9:**
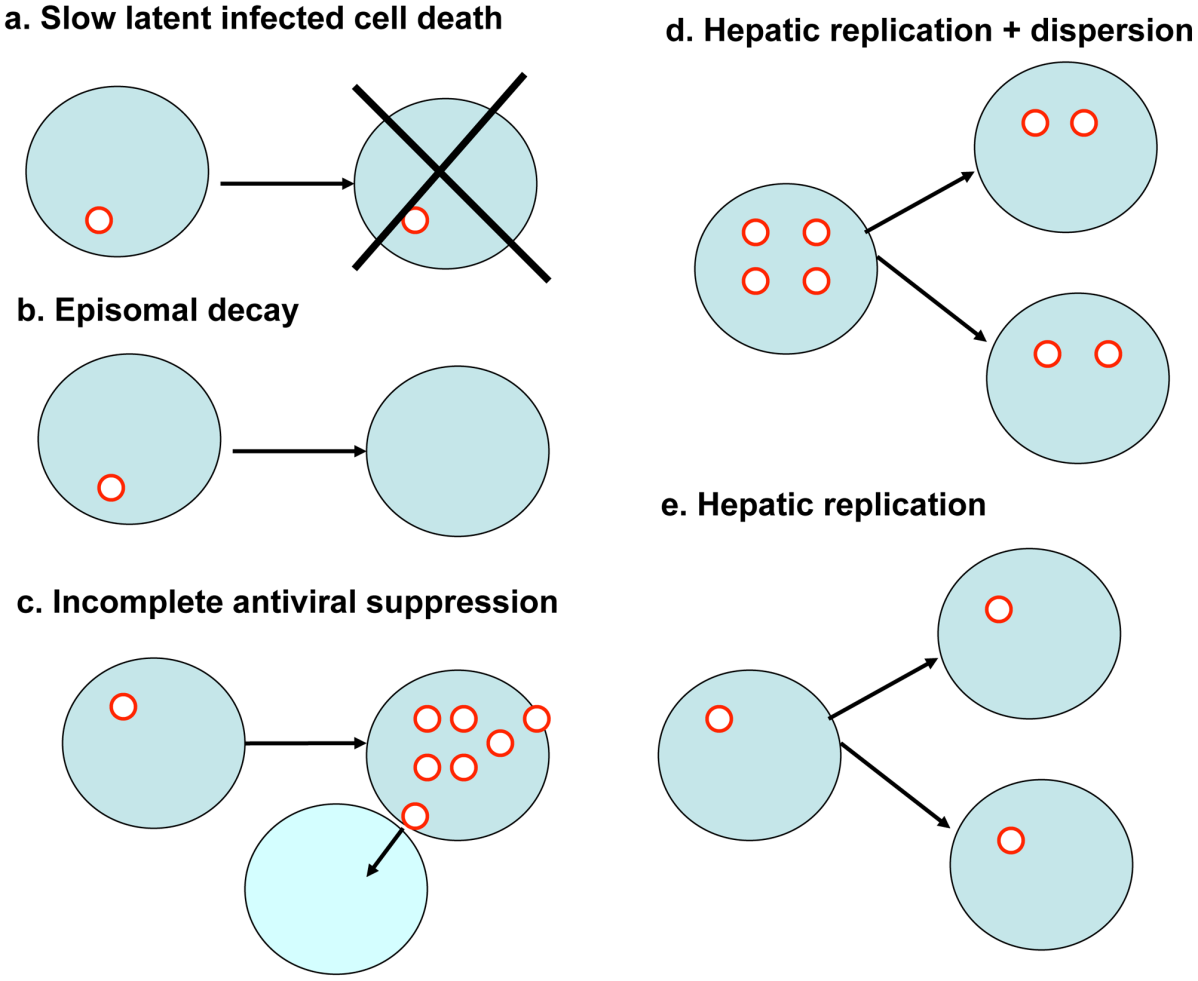
Possible dynamics of HBV cccDNA between vector delivery doses. (a) Cell death inducing decay of incorporated episome. (b) Episomal degradation. (c) cccDNA expansion despite suppressive antiviral therapy. (d) Hepatocyte replication with equal dispersion of cccDNA molecules between cells. (e) Hepatocyte replication with replication of cccDNA molecules between cells. Only mechanism (c) could increase number of doses needed prior to inactivation while other mechanisms (a, b and d) may allow more rapid cure.

If cccDNA levels reconstitute at a meaningful rate and several day intervals are required between doses to allow effects of DNA cleavage enzymes to accrue, then this may imply the need for more prolonged therapeutic courses. We therefore examined the effects of underlying dynamics of HBV cccDNA survival, as well as the possible delayed effects of DNA cleavage enzymes following target cell entry. Several factors may drive changes in levels of HBV cccDNA during suppressive antiviral therapy. Hepatocytes with HBV cccDNA molecules periodically die at a rate equivalent to that of an uninfected hepatocyte (**Fig. 9a**). Decay of individual episomes at a slow rate is possible (**Fig. 9b**) but has not been explicitly documented and may be counterbalanced by low-level replication despite antiviral therapy, which may also allow spread to uninfected cells (**Fig. 9c**). Indeed, many patients do not achieve full virologic suppression [6]. Finally, most evidence supports division of nuclear cccDNA between daughter cells during homeostatic proliferation (**Fig. 9d**) [33]: as a result, patients on antiviral therapy appear to have a slow decay in levels of cccDNA over time though this decline is not rapid enough for viral eradication [58]. A less optimistic assumption for the standpoint of achieving cure would be that episomes divide along with human chromosomal DNA during cell division, limiting cccDNA decay (**Fig. 9e**).

**Figure 10:**
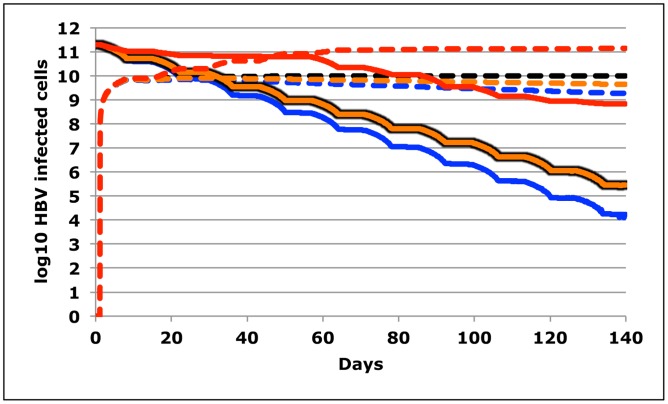
Underlying HBV cccDNA dynamics are unlikely to have a significant outcome on therapeutic outcome. Simulation of HBV inactivation following 10 doses of therapy given every two weeks. Unlike prior simulations, these simulations assume that DNA cleavage activity accrues evenly over a week rather than instantaneously. Parameters reflect high potency (m*σ = 5, d = 0.004, h = 2). Traces represent no cccDNA activity between does (black lines), residual cccDNA replication between doses at rate = 0.01/day (red lines), hepatocyte replication with dispersion of cccDNA between cells (orange lines) and hepatocyte death with concurrent death of episomes (blue lines). Solid lines are cells with susceptible episomes and would represent therapeutic outcomes in the absence of de novo resistance (Ψ = 0). Dotted lines are cells with resistant episomes and would represent therapeutic outcomes with de novo resistance (Ψ = 0.01). Effects on therapeutic outcomes are minimal unless fairly high levels of cccDNA turnover are assumed (red lines) as may occur in the absence of fully suppressive antiviral therapy.

In all simulations in **Fig. 10**, enzyme dosing occurred every two weeks. However, enzyme activity was assumed to accrue continuously over a week rather than instantaneously. We first assumed baseline conditions with high potency (**Fig. 10**) with no change in cccDNA levels between doses. A simulation with homeostatic proliferation of cccDNA (**Fig. 9d**), revealed a marginally lower level of remaining viral episomes after 10 doses, while episomal death concurrent with hepatocyte death (**Fig. 9a**) augmented episomal decay more substantially. If poor control of cccDNA replication was assumed due to incomplete suppression by antiviral drugs (**Fig. 9c**), then therapy was less potent.

## Discussion

We describe mathematical models that aim to capture critical features of DNA cleavage enzyme therapy for eradication of HBV. Our results identify potentially critical parameters that will determine whether cure will be feasible with available vector cleavage enzyme constructs. In particular, successful vector delivery to the majority of target cells with each infusion, and favorable intracellular binding kinetics between enzymes and DNA target sites appear to be pre-requisites for successful regimens. Cooperative binding of enzymes between multiple episomal targets could also potentially limit the number of doses needed prior to cure, particularly if enzyme concentration in cells only marginally exceed binding coefficient values.

While multiple doses of gene therapy will likely be required for cure, the first dose appears to be particularly critical. In order to enhance potency and limit resistance, this dose should have a high vector to target cell ratio, and if possible, multiple enzymes should be packaged within each delivery vector. Sequential use of different enzymes appears to be another useful strategy to avoid *de novo* resistance if only low-payload delivery vectors such as AAV are available.

While our integrated therapeutic model is relatively complex, its individual components (vector delivery, intracellular pharmacodynamics, resistance) are quite manageable. In total, the model contains only five unknown parameter values including 1) proportion of vectors removed prior to entry into target cells, 2) enzyme-DNA binding coefficient, 3) vector-DNA cleavage dose response slope (Hill coefficient), 4) resistance rate per DNA cleavage event and 5) dose response slope within a single episome if multiple enzymes are present in the cell nucleus. Each of these parameter values can be identified via specific experimental approaches for all vectors and cleavage enzymes of interest, which will allow for testing and refining of the model.

Vector delivery to target cells is best estimated initially in animal model studies. Humanized mouse models of HBV hold promise for this indication [59], [60]. Flow cytometry of liver biopsy tissue can be employed to quantify proportion of target cells without vector delivery following different doses of vector; the effective multiplicity of infection (σ*m) can be back calculated using P_v_(0) = [(σ*m)^v^ * e^−(σ*m)^]/v!. This effective delivery dose will represent a fraction of the pre-determined vector to target cell ratio (m), which in turn will allow for an estimate of proportion of vectors lost prior to target cell entry (1−σ). Ultimately, these experiments will need to be conducted in humans, as the human immune response to delivery vectors cannot be predicted from animal models. However, animal model parameters will serve as useful initial estimates that may be used within a Bayesian framework to assist in human clinical trial design.

A critical caveat of the functional MOI (fMOI) is that the vector to target cell ratio assumed in parameter m is inclusive of all cells that may serve as targets for vector entry, rather than only HBV infected cells. If a particular delivery vector also efficiently enters other intrahepatic cells such as Kupffer cells, endothelial cells or cells in other organs, then the fMOI will decrease accordingly. In effect, these cells will serve as vector sponges and will decrease the probability of high vector delivery to infected cells containing HBV cccDNA. Therefore, vector receptor specificity is critical not only to avoid untoward toxicity, but also to ensure that precious vector is not wasted.

A key experimental goal should be to determine which enzymes achieve avid binding and DNA cleavage activity (low values for d) and positive cooperative binding (h>1 or z>1) to their DNA targets. Dose response curve slope and enzyme-substrate binding coefficients can be obtained from cell culture models of HBV cccDNA infection in which infected cell lines are exposed to delivery vectors dosed at different multiplicities of infections. Using high throughput sequencing of the DNA target site, it will be possible to measure the proportion of target genomes with terminally disrupted DNA for each vector dose. Experimental dose response curves can be tested against our models describing enzyme DNA binding kinetics. If multiple enzymes are delivered concurrently in a single vector, then similar curves can be used to assess cooperative binding between several sites within a single episome.

To obtain a conservative upper limit for resistance rate per cleavage event, it will first be necessary to identify cells with confirmed vector delivery and HBV cccDNA cleavage. One possibility is to sort for vector transduced cells that are HBV e antigen positive and then look for mutation events within the cleaved open reading frame. For the purposes of informing clinical trial dose design, this estimate will be useful to ensure that doses exceed predicted thresholds for viral persistence.

When all unknown parameter values are estimated and a model structure is selected that best represents available data regarding vector delivery, enzyme/DNA substrate kinetics and resistance rate, then it will be possible to design regimens that maximize probability of cure while limiting excess dosing and possible toxicity. While it will be necessary to characterize all available delivery vectors and cleavage enzymes prior to predicting likelihood of therapeutic success, certain strategies are promising based on *in silico* simulations. For instance, if multiple transgenes targeting different viral DNA regions can be packaged within the same delivery vector, at least during the first dose, this may augment potency and decrease resistance when compared to multiple transgenes split among vectors. Ensuring high delivery during the first dose will maximize this effect.

A key challenge will be measuring therapeutic outcome. For HBV, it is difficult to take serial quantitative measures of episomal reservoirs of infection. While active viral replication can be tracked with quantitative PCR, burden of quiescent viral episomes can only be assessed with liver biopsy and tissue quantitation of uncleaved HBV cccDNA using sequencing. Even a tiny number of latently infected cells may theoretically be enough to reactivate and repopulate the reservoir. Because serial biopsies are likely to be feasible only in animal models of infection, therapeutic efficacy will ultimately need to be evaluated with close clinical follow up after cessation of antiviral therapy. For this reason, we make conservative assumptions in our model, so that the dosing schedule exceeds the presumed threshold for cure.

While we have focused on eradication of HBV, our model is easily adjusted to account for potential cure of other chronic viral infections such as HIV or HSV-2. The burden and properties of non-replicating viral stores differ dramatically between HBV, HIV and HSV [10]. As such, each infection presents a unique set of challenges for eradicative approaches. While latent HIV integrates as viral DNA into the human genome, HIV-1 DNA is present in only ∼10^7^ cells during chronic infection, typically as a single genome per cell [61], [62]. However, the HIV-1 reservoir may be anatomically difficult to target with delivery vectors; while memory CD4+ T-cells are the central population of cells within the latent reservoir, the possibility that other immune cells form important reservoirs has not been completely excluded and if target receptors on these cells differ, then they may serve as sanctuaries from therapeutic cleavage enzymes [63]. Finally, due to rapid HIV-1 intra-host evolution in the context of ongoing immunological pressure, the HIV-1 reservoir is populated with diverse quasispecies, which may lead to pre-existing resistance to certain cleavage enzymes [64]. Therefore, phylogenetic techniques may be necessary to explore for bottleneck effects if a majority, but not all viral strains, are eliminated following repeated dosing of DNA cleavage enzymes.

HSV latency exists within a relatively low number of neuronal cell bodies in either the trigeminal or dorsal root ganglia [56], which may represent a therapeutic sanctuary where delivery of vectors is poor. For HSV-2, sampling of the dorsal root ganglia, the site of latency, is not feasible. Close clinical follow up following gene therapy will be necessary to evaluate for cure. As with HIV-1, the possibility of re-infection will need to be considered using phylogenetic sampling of pre and post-treatment positive PCR samples, as inactivation may not ensure protective immunity from re-exposure.

In summary, we present a model to capture the effects of gene therapy with DNA cleavage enzymes for chronic HBV infection. The model helps identify key therapeutic parameters that will be necessary for cure, and outlines appropriate experimental steps to identify dosing regimens that are most likely to disrupt all latent viral DNA following a minimal number of gene therapy doses.

## Methods

Simulations were performed on C++ and using Microsoft Excel.

## Supporting Information

Movie S1 **Simulation with weekly dosing of HBV DNA cleavage enzymes.** The number of cells with a given combination of susceptible remaining genomes (x-axis) and resistant remaining genomes (y-axis) is displayed following each dose. The goal of an eradicative therapy is for all cells to enter the top left box (no sensitive or resistant viral genomes). This simulation of a potent regimen with high fMOI (m*σ = 5), high enzyme – DNA binding avidity (d = 0.04), and positive binding cooperativity (h = 2), as well as high resistance rate (0.05), leads to >10^10^ cells harboring 1 or more enzyme sensitive genome, >10^9^ cells harboring one resistant genome only and >10^8^ cells harboring 2 or more resistant genomes only at 140 days (or 20 doses) following initiation of therapy.
(WMV)
